# Novel probiotic lactic acid bacteria isolated from indigenous fermented foods from West Sumatera, Indonesia

**DOI:** 10.14202/vetworld.2020.1922-1927

**Published:** 2020-09-19

**Authors:** Harnentis Harnentis, Yetti Marlida, Yuliaty Shafan Nur, Wizna Wizna, Melia Afnida Santi, Nadia Septiani, Frederick Adzitey, Nurul Huda

**Affiliations:** 1 Department of Animal Nutrition and Feed Technology, Faculty of Animal Science, Andalas University, West Sumatera, Indonesia; 2 Department of Animal Nutrition, Faculty Animal Husbandry, Universitas Muhammadiyah Tapanuli Selatan, North Sumatera, Indonesia; 3 Department of Veterinary Science, Faculty of Agriculture, University for Development Studies, Box TL 1882, Tamale, Ghana; 4 Department of Food Science and Nutrition, Faculty Food Science and Nutrition, Universiti Malaysia Sabah, 88400, Kota Kinabalu, Sabah, Malaysia; 5 Department of Food Technology, Faculty of Agriculture, Universitas Sultan Ageng Tirtayasa, Banten 42124, Indonesia

**Keywords:** fermentation, lactic acid bacteria, poultry, probiotic

## Abstract

**Background and Aim:**

Probiotics play an important role in maintaining a healthy gut and consequently promote good health. This study aimed to find novel probiotic lactic acid bacteria (LAB) from indigenous fermented foods of West Sumatera, Indonesia.

**Materials and Methods:**

This study utilized 10 LAB previously isolated from fermented buffalo milk (*dadih)*, fermented fish (*budu*), and fermented cassava (*tape*) which have the ability to produce gamma-aminobutyric acid. The study commenced with the screening of LAB for certain properties, such as resistance to acid and bile salts, adhesion to mucosal surface, and antagonism against enteric pathogens (*Escherichia coli, Salmonella* Enteritidis, and *Staphylococcus aureus*). The promising isolates were identified through biochemical and gram staining methods.

**Results:**

All isolates in this study were potential novel probiotics. They survived at a pH level of 2.5 for 3 h (55.27-98.18%) and 6 h (50.98-84.91%). Survival in bile at a concentration of 0.3% was 39.90-58.61% and the survival rate was 28.38-52.11% at a concentration of 0.5%. The inhibitory diameter ranged from 8.75 to 11.54 mm for *E. coli*, 7.02 to 13.42 mm for *S. aureus*, and 12.49 to 19.00 mm for *S*. Enteritidis. All the isolates (84.5-92%) exhibited the ability to adhere to mucosal surfaces. This study revealed that all the isolates were potential probiotics but N16 proved to be superior because it was viable at a pH level of 2 (84.91%) and it had a good survival rate in bile salts assay (55.07%). This isolate was identified as *Lactobacillus* spp., Gram-positive bacilli bacteria, and tested negative in both the catalase and oxidase tests.

**Conclusion:**

All the isolates in this study may be used as probiotics, with isolate N16 (*Lactobacillus* spp.) as the most promising novel probiotic for poultry applications based on its ability to inhibit pathogenic bacteria.

## Introduction

Indigenous fermented foods in West Sumatera, Indonesia, that are naturally fermented with or without adding microbes or inoculum include *dadih, tape*, and *budu*. These foods are very popular in the regions of Padang, Bengkulu, Riau, Jambi, Lampung, and Aceh. They have a distinctive smell, sour taste, and are creamyand yellow in color. Fermentation can improve the storage life and taste of *dadih, tape*, and *budu*. In West Sumatera, fermented foods are produced on a small scale as part of household businesses, and the fermentation processes are traditional and are influenced by local culture. Traditional Indonesian fermented foods can be used as potential sources of probiotics as they commonly contain lactic acid bacteria (LAB), including species of *Lactobacillus*, *Pediococcus*, *Enterococcus*, *Weissella*, and *Leuconostoc* [1]. These are effective in inhibiting the growth of pathogenic organisms through different mechanisms, such as adherence to epithelial cells and immune system modulation [2]. Palachum *et al*. [3] stated that a potent probiotic isolate must possess certain characteristics, such as survival and colonizing ability under different environmental conditions. The isolates should be able to withstand the low pH of gastric juice, be resistant to bile salts, and should adhere to epithelial cells [4].

In the period between 2017 and 2019, Marlida, together with her doctoral student Anggraini, conducted research on the isolation of LAB from local fermented foods in West Sumatra, Indonesia. The fermented foods were *dadih*, *tape*, and *budu*, and they found 704 isolates of LAB. Anggraini *et al*. [5] then screened 704 isolates of LAB for their ability to produce gamma-aminobutyric acid, which is useful as a feed additive and as anti-heat stress for broilers. Anggrainia *et al*. [6] identified 10 potential isolates. LAB from fermented foods can potentially be developed as probiotics for livestock, especially poultry, since probiotics for poultry are usually isolated from the digestive tract of livestock. The gut is a potential source of LAB, as well as a potential source of probiotics [6]. Reuben *et al*. [7] added that *Lactobacillus reuteri* I2, *Pediococcus acidilactici* I5, *P*. *acidilactici* I8, *P*. *acidilactic*i c3, *P. pentosaceus* I13, and *Enterococcus faecium* c14 were LAB with probiotic potential isolated from the growth inhibition test (GIT) of apparently healthy broiler chickens (with 35 and 22 from the intestine and crop, respectively). Hidayat *et al*. [8] isolated and characterized LAB from 3-day-old broilers and found *Enterococcus* and *Lactobacillus*.

The fermentation process can occur naturally because of the microbes that are already present in foods when they grow with or without the addition of microbial cultures, and the latter produces a more uniform product [5]. Some researchers have reported on the isolation of probiotics from fermented products, such as traditionally fermented Ethiopian food products [9], sap extract of the coconut palm inflorescence – Neera, which is a naturally fermented drink consumed in various regions of India [4], and Chinese fermented food products [10].

The ability of LAB to be probiotic varies according to where they were isolated. Kim *et al*. [11] isolated four types of LAB from different specimens and found that their probiotic properties differed in terms of resistance to gastric pH and bile acids, but were similar in terms of reducing pig manure odor. Fermented foods originating from different countries will certainly produce LAB that have different properties, especially as probiotics. Pathogenic microorganisms include some species of *Escherichia coli, Salmonella* Enteritidis, and *Staphylococcus aureus*. These pathogens can colonize the gastrointestinal tract of poultry and contaminated poultry carcasses under faulty living conditions. Subsequently, they can be a source of infections for humans. The use of probiotics to control or reduce the number of these pathogens in the gastrointestinal tract of poultry is, therefore, essential to reduce transmission of infections to humans.

This study aimed to find novel probiotics for poultry from LAB isolated from indigenous fermented food from West Sumatera, Indonesia.

## Materials and Methods

### Ethical approval

This study did not use human or animal subjects, and as such ethical clearance was not required.

### Study period and location

This study was conducted at Feed Processing Laboratory of the Department of Animal Nutrition and Feed Technology, Faculty of Animal Science, Andalas University, West Sumatera, Indonesia, from January 2019 to June 2019.

### Sources of lactic acid bacteria

The study utilized a descriptive method by testing the ability of LAB as probiotic candidates based on several parameters. There were 10 isolates included in this study (N40, N16, N32, N1, C33, C16, and B48 of *dadih* origin, P1 and P15 of *tape* origin, and L3 of *budu* origin). The isolates were recovered from storage using De Man, Rogosa, Sharpe (MRS) broth. For the recovery, a 1 ml suspension of the isolates was transferred into 10 ml MRS broth and incubated at 37°C for 24 h [5].

### Test for gastric pH

Gastric pH testing was based on the modified method from Dowarah *et al*. [12]. This test was performed using 10 LAB isolates. Two types of MRS broth were used. One was mixed with 37% HCl to obtain a pH of 2.5 and the other served as the control. Thereafter, 0.5 ml containing 10^9^ CFU/ml of bacterial isolates were transferred into 5 ml MRS-Hydrochloric acid or MRS broths and incubated for 6 h at 37°C. The absorbance was read at a wavelength of 600 nm. This research was replicated 3 times. Resistance to gastric pH was expressed in percentages according to the standards set by Tokatli *et al*. [13].

### Test for resistance to bile salt

The bile acid resistance test was based on the modified method from Nwachukwu *et al*. [14]. The test was performed by adding bile salt (oxgall) at 0%, 0.3%, and 0.5% to MRS broth. After this, 0.5 ml (10^9^ CFU/ml) of bacterial suspension was added to 5 ml of the MRS broth and incubated at 37°C for 5 h. Controls in the MRS broth without the addition of bile acid (0% bile acid) were compared to the treatment group. Growth was measured at a wavelength of 600 nm. Resistance to bile salt was expressed in percentages.

### Inhibition test against pathogenic bacteria

Antimicrobial activity of the LAB against *E. coli*, *S*. Enteritidis, and *S. aureus* was measured based on a modified method from Bagis *et al*. [15]. Briefly, blank antibiotic disks were soaked in a LAB suspension for 10 min. These were then transferred into nutrient agar, which had its surface spread plated with either *E. coli*, *S*. Enteritidis, or *S. aureus*. After that, the nutrient agar plates were incubated at 37°C for 24 h, and the diameter of the inhibition zones was measured using calipers.

### Hydrophobicity of LAB on stainless steel plates

The hydrophobicity test or attachment test was performed using a modified method from El-Jeni *et al*. [16]. Briefly, LAB were cultured in sterile MRS broth overnight. Thereafter, the bacterial culture (500 μl) was transferred into a test tube, filled with 450 μl of MRS broth, wherein the sterile stainless steel plate was deposited, and the test tubes were then incubated for 24 h at 37°C. The stainless steel plates were removed under aseptic conditions, washed with 10ml of sterile 1% peptone water, and left for 5 min in a sterile 1% peptone water tube. The plate was then washed again in the same conditions and vortexed for 3 min in a sterile 1% peptone water tube (6 ml) consecutively to detach the bacterial cells adhering to the steel plate surface. The cell number was determined by counting on the MRS agar after 24 h of incubation at 37°C. Simultaneously, the total initial cell numbers were estimated to calculate the percentage bacterial cell adhesion for each LAB.

## Results and Discussion

### Resistance of LAB to stomach pH

Testing the resistance of LAB against gastric pH was carried out at a pH of 2.5, because the pH of the proventriculus and gizzard is between 2.5 and 3.5 [17]. It is also within the pH range at which digestive enzymes are secreted and functions in the proventriculus, to bring about digestion of proteins, carbohydrates, and other food substances, and it has the longest food transit time (90 min) compared to other parts of the digestive system. The proventriculus is the true stomach compartment in birds, where hydrochloric acid and pepsinogen are secreted by the proventriculus and mixed with contents through the peristalsis of the gizzard [12].

The results obtained after 3 and 6 h of incubation are shown in Table-1. The results from Table-1 show that all the LAB at 3 or 6 h of incubation can survive at a pH of 2.5 with a minimum resistance of ≥50%, which means that all the LAB in this study can be utilized as probiotics. These results were also reported by the study conducted by Mulaw *et al*. [9], where the percentage survival of LAB against pH 2.5 for 3 h was >50%. As shown in Table-1, the isolate which had the highest resistance was N16. N16 was isolated from fermented buffalo milk (*dadih*) with a strong resistance of 88.80% at an incubation time of 3 h, and this resistance was decreased at an incubation time of 6 h to 84.91%; thus, only an incremental decrease occurred (3.89%). A small decrease translates to a higher survival rate. This is consistent with the findings of Nurnaafi *et al*. [18], which showed that probiotics have a higher survival rate and a small decrease in growth rate. Thus, any LAB with these characteristics can be considered as probiotic. This study yielded higher results compared to those of Mulaw *et al*. [9], who found that LAB isolated from traditionally fermented Ethiopian food products (*Teff* dough, *Ergo*, and *Kocho*) had a survival rate of 90.13% at a pH of 2.5 and with an incubation period of 2 h. Tokatli *et al*. [13] reported that *Lactobacillus brevis*, *Lactobacillus plantarum*, and *Pediococcus ethanolidurans* isolated from traditional pickles had a survival rate of 33-64%, 35-85%, and 40-76%, respectively, at a pH of 2.5 and an incubation period of 4 h. Furthermore, a survival rate of ≥80% at a pH of 2.5 and an incubation period of 4 h was observed for *Lactobacillus fermentum* isolated from fermented milled flour [19].

**Table-1:** Resistance of lactic acid bacteria to gastric pH (%).

LAB isolates	Time (3 h)	Time (6 h)
N40 (*dadih* origin)	55.27±0.85	50.98±1.26
N16 (*dadih* origin)	88.80±4.34	84.91±0.22
N32 (*dadih* origin)	98.18±0.64	77.11±0.39
N1 (*dadih* origin)	87.03±4.51	70.29±3.64
C33 (*dadih* origin)	85.32±0.74	76.80±0.74
C16 (*dadih* origin)	71.06±1.36	55.42±2.68
B48 (*dadih* origin)	98.48±1.06	77.70±4.11
L3 (*budu* origin)	85.52±2.60	51.84±0.83
P1 (*tape* origin)	88.74±2.52	82.12±0.84
P15 (*tape* origin)	86.50±2.52	51.87±1.68

±=Standard deviation, n=3

Probiotic LAB candidates should be capable of withstanding the extreme conditions in the digestive tract, from the mouth to the intestines, and should be able to subsequently colonize the intestinal surface. According to Evivie *et al*. [20], gastric acidity serves as a precondition prior to conducting microbial selection before entering the intestines. The acid resistance of LAB is of great importance not only for their own growth but also for the fermentation and preparation of probiotic products [21]. Several mechanisms are involved in the acid resistance regulation of LAB, including central metabolic pathways, proton pumps, changes in cell membrane composition and cell density, DNA and protein damage repair, as well as neutralization processes [22,23].

The cell wall of Gram-positive bacteria is made up of 90% peptidoglycan and thin layers of teichoic acid (TA) [24]. TA is the main component of the cell wall of Gram-positive bacteria. TA is composed of glycerol or ribitol chains connected by phosphoric acid and phosphodiester bridges [25]. Peptidoglycan is made up of mainly N-acetylmuramate and N-acetylglucosamine, which are derivatives of sugar, and several amino acids such as D-alanine, L-alanine, D-glutamic acid, and diaminopimelic acid. TA contains glycerol or ribitol units, which are bound by phosphate esters and contain other sugars and D-alanine. This thick peptidoglycan, along with the chains of TA, can maintain the shape of the cell wall even in acidic extracellular conditions. Acidic extracellular conditions can cause lysis of LAB cell walls, but the cell walls can maintain their shape to protect cellular contents. The lipid layer, which is thinner, causes the pores of the walls to shrink so that cell permeability is reduced and the extraction of intracellular components by acid cannot damage the lipid layer that is on the cell membrane. However, this lipid layer contains special proteins; some of the membrane proteins are enzymes, while others can bind to nutrients and transport them into the cells [7].

### Resistance of LAB to bile salt

LAB resistance to 0.3% and 0.5% bile salts (oxgall) is presented in Table-2. The criteria for LAB to be considered as probiotic include resistance to bile salts as well as resistance to acidic conditions. Bile tolerance is one of the most crucial properties for probiotic bacteria to have, as it determines the bacteria’s ability to survive in the small intestine and consequently its capacity to play its functional role as a probiotic [9].

**Table-2:** Resistance of lactic acid bacteria to 0.3-0.5% bile salts.

LAB	Resistance (%)	

	0.3% bile salt	0.5% bile salt
N40	46.65±0.42	34.09±1.31
N16	55.07±0.80	47.45±1.08
N32	58.61±1.49	52.11±1.12
N1	46.72±1.24	36.88±0.56
C33	57.95±1.55	49.04±0.55
C16	49.83±0.79	31.56±2.72
B48	55.31±1.89	45.72±2.08
L3	42.18±1.48	36.31±0.23
P1	42.41±1.02	30.80±0.64
P15	39.90±1.06	28.38±1.59

±=Standard deviation, n=3

The ability of a potential probiotic strain to tolerate or withstand intestinal bile salt is of immense importance to their survival and growth in the GIT; thus, it is a major requirement for probiotic selection. In the poultry GIT, the duodenum and cecum have a total bile salt concentration of 0.175 and 0.008% [7], and the bile salt concentration for humans’ ranges from 0.14 to 0.93 mM [26]. However, the average level of 0.3% bile salt has been considered in many studies as the threshold for bile salt tolerance of a potential probiotic [9]. In our study, all the LAB strains examined were able to tolerate 0.3% bile salt after 6 h of incubation (Table-2).

The results presented in Table-2 show that all the isolates of LAB in this study can withstand bile salts with a resistance of >30%. According to Bustos *et al*. [27], LAB can withstand bile salts with a resistance of 20-40%. Nurnaafi *et al*. [18] added that good probiotic candidates are isolates that have survival rates of more than 50% under low pH conditions and are resistant to bile salts. In this study, four of the LAB isolates were considered as good probiotic candidates as they had resistances of ≥50% at a concentration of 0.3% bile salt.

N32 had the highest resistance of 58.61% in 0.3% bile salt. When the concentration of bile salt was increased to 0.5%, the resistance decreased to 52.11% (a percentage reduction of 3.5%). A small percentage decrease translates to a higher survival rate. This is consistent with the findings of Nurnaafi *et al*. [18], who stated that LAB that are considered as probiotics have high survival rates. N16 also showed good resistance second to N32. At a bile salt concentration of 0.3%, the resistance was 55.07% and when the concentration of bile salt was increased to 0.5%, the resistance decreased to 47.45% (a percentage reduction of 7.62%). This result was higher than those found by Melia *et al*. [28], who reported that LAB isolated from buffalo milk can survive in 0.3% and 0.5% bile salts after 5 h of incubation with a rate of 40.58% and 35.22%, respectively. Guan *et al*. [29] reported that *L. plantarum* (HLX37) isolated from fermented milk survived well in 0.3% bile salts at a rate of 54.68% and at an incubation period of 4 h.

An important characteristic for LAB to be considered as probiotics is its ability to resist bile salts in the small and large intestines [30]. The results of this study indicated that the identified LAB can survive in small and large intestines. According to Bustos *et al*. [27], LAB are able to survive in bile salts because they contain bile salt hydrolase (BSH), an enzyme which is active in the form of bile acids. Bacterial membranes are the main targets of bile acids. For bacteria to survive bile salts, they produce BSH by conjugation into free bile acids. Free bile acids can participate in a variety of metabolic processes, including the regulation of fat absorption; cholesterol metabolism; the creation of homeostatic conditions in the bacterial membrane; and regulating nitrogenous bases, fats, and amino acid biosynthesis, which allow changes in fat, resulting in exopolysaccharide (EPS) production. EPS functions as protective agents against bile salts (0.15-0.3%) at a pH of 2-3 [31].

### Inhibition of LAB to pathogenic bacteria

The inhibition of LAB against pathogenic bacteria (*E. coli*, *S. aureus*, and *S*. Enteritidis) is presented in Table-3. These pathogenic bacteria, such as *E. coli*, *S. aureus*, and *S*. Enteritidis, are bacteria that can kill broilers or cause foodborne diseases in humans. These results are presented in Table-3, showing that N16 exhibited the highest inhibition of LAB isolates against *E. coli*, with an inhibition zone of 11.54 mm. Ren *et al*. [32] found that *Lactobacillus* T8 produced antibacterial substances belonging to a protein family, and its zone of inhibition against pathogens significantly increased (>13 mm) after these substances were produced. Obdak *et al*. [33] added that *L. plantarum* strains showed strong antimicrobial activities against a wide range of potential pathogens, especially *Listeria monocytogenes*. Thus, *L*. plantarum can be considered as a good probiotic candidate for extending the lifespan of fermented foods.

**Table-3:** Inhibitory diameter against pathogenic bacteria.

LAB	Inhibition zone (mm)		

	*Escherichia coli*	*Staphylococcus aureus*	*Salmonella* Enteritidis
N40	10.01	7.02	17.39
N16	11.54	10.27	16.31
N32	8.78	10.09	13.23
N1	9.55	7.46	13.38
C33	9.06	11.83	14.81
C16	9.15	13.42	19.00
B48	10.83	8.51	16.33
L3	8.93	9.63	18.63
P1	9.82	11.65	13.93
P15	8.85	9.30	12.49

±=Standard deviation, n=3

Damage to the cellular membrane of pathogenic bacteria retards their metabolic processes and prevents growth because lactic acid diffuses into the bacterial cell and can disrupt the integrity of the cell membrane [7]. According to Mulaw *et al*. [9], several types of LAB can produce bacteriocin, which is an antibacterial peptide and a protein-containing toxin that can prevent bacterial growth. LAB produces an acidic environment and bacteriocin which increases its ability of to stop the growth of harmful bacteria andother competing bacteria [27].

The 10 LAB in this study had different inhibitory zones against pathogenic bacteria. This is due to the fact that LAB have different properties used to destroy pathogens. LAB can be heterofermentative or homofermentative. Homofermentative LAB produce only organic acids, while heterofermentative LAB produce organic acids and antimicrobial compounds. Thus, heterofermentative LAB have the ability to destroy highly pathogenic bacteria. In this study, LAB produced larger inhibition zones against *E. coli*, *S*. Enteritidis, and *S. aureus*; therefore, they were heterofermentative. In addition, the pathogenicity of bacteria affects the ability of LAB to destroy them. The ability of Gram-positive pathogenic bacteria to resist destruction by LAB is higher than that of Gram-negative bacteria. This is due to the fact that Gram-positive bacteria have peptidoglycan and TA, which makes up 90% of the cell wall.

### Hydrophobicity of LAB

**Figure-1:**
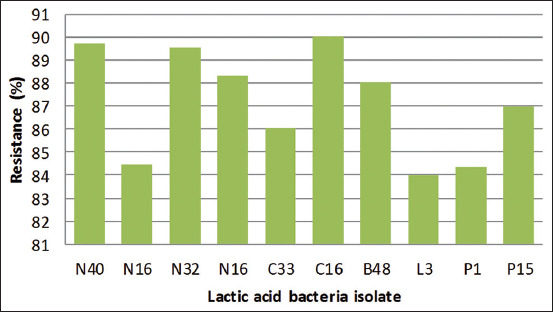
Hydrophobicity of LAB.

The results presented in Figure-1 show that all the LAB isolates had >84% hydrophobicity ability. C16 had the highest hydrophobicity ability, which was 90.01% while L3 had the least hydrophobicity, which was 84.02%. The hydrophobicity of the LAB included in this study was higher than the findings of Mulaw *et al*. [9], who found that a LAB strain, *Lactobacillus* spp., isolated from traditionally fermented Ethiopian food products, had a hydrophobicity ability of 32.75-36.30%. However, research conducted by Tokatli *et al*. [13] revealed that the LAB strain, *L. plantarum*, which was isolated from traditional pickles, had a hydrophobicity ability of 82.41%.

A criterion for determining which LAB can be considered as probiotic is the bacteria’s capacity to attach itself to the epithelium of the intestinal mucosal [25]. Hydrophobicity is related to the presence of cell wall components, such as phospholipids, polysaccharides, and other external components on the bacterial cell surface [34]. The presence of proteins and lipoteichoic acids on cell surfaces provides cells with hydrophobic properties, whereas the presence of polysaccharides produces hydrophilic properties [24]. Adhesion ability is a primary criterion for the selection of probiotic microorganisms. LAB constitutes the majority of microorganisms with probiotic properties. They have several important mechanisms for intestinal epithelial cell adhesion. They generally use various structures to adhere to intestinal cells, such as flagella, pili, S-layer proteins, lipoteichoic acid, EPSs, and mucus-binding proteins [25]. All the LAB in this research yielded negative results on the blood agar test, which involves hemolysis. Thus, the LAB in this study are non-pathogenic when judged according to the criteria set by Anggraini *et al*. [5].

## Conclusion

*In vitro* testing of the 10 LAB isolates to determine which strains are probiotic revealed that N16, isolated from *dadih*, had a survival rate of 88.80% at a pH level of 2.5 for 3 h. At 6 h, survival rate was 84.91%; and in 0.3% and 0.5% bile salts, it was 55.07% and 47.45%, respectively. With regard to its ability to eradicate pathogens, it produced inhibition zones of 16.31 mm for *S*. Enteritidis, 11.54 mm for *E. coli*, and 10.27 mm for *S. aureus*. N16 was found to be Gram-positive bacilli bacteria, catalase and oxidase negative, and was classified as *Lactobacillus* spp.

## Authors’ Contributions

HH, YM, YSN, WW, MAS and NS collected data and wrote the manuscript. YM designed the study. FA and NH reviewed and updated the manuscript. All authors read and approved the final manuscript.
